# Acid Free Oxidation and Simple Dispersion Method of MWCNT for High-Performance CFRP

**DOI:** 10.3390/nano8110912

**Published:** 2018-11-06

**Authors:** Gerald Singer, Philipp Siedlaczek, Gerhard Sinn, Harald Rennhofer, Matej Mičušík, Maria Omastová, Miriam M. Unterlass, Josef Wendrinsky, Valeria Milotti, Filippo Fedi, Thomas Pichler, Helga C. Lichtenegger

**Affiliations:** 1 Institute of Physics and Materials Science, University of Natural Resources and Life Sciences Vienna, Peter-Jordan-Straße 82, 1190 Vienna, Austria; gerald.singer@boku.ac.at (G.S.); philipp.siedlaczek@boku.ac.at (P.S.); gerhard.sinn@boku.ac.at (G.S.); harald.rennhofer@boku.ac.at (H.R.); 2 Slovak Academy of Sciences, Polymer Institute, Dúbravská cesta 9, 845 41 Bratislava 45, Slovak Republic; upolmmic@savba.sk (M.M.); upolmaom@savba.sk (M.O.); 3 Institute of Materials Chemistry, Technische Universität Wien, Getreidemarkt 9/165, 1060 Vienna, Austria; miriam.unterlass@tuwien.ac.at (M.M.U.); josef.wendrinsky@aon.at (J.W.); 4 Institute of Applied Synthetic Chemistry, Technische Universität Wien, Getreidemarkt 9/163, 1060 Vienna, Austria; 5 Electronic Properties of Materials, Universität Wien, Boltzmanngasse 5, 1090 Vienna, Austria; valeria.milotti@univie.ac.at (V.M.); filippo.fedi@univie.ac.at (F.F.); thomas.pichler@univie.ac.at (T.P.)

**Keywords:** oxidation, functionalization, eco-friendly, dispersion, carbon fiber reinforced polymer (CFRP), mechanical properties

## Abstract

Carbon nanotubes (CNT) provide an outstanding property spectrum which can be used to improve a wide range of materials. However, the transfer of properties from the nanoscale to a macroscopic material is a limiting factor. Different approaches of functionalizing the surface of a CNT can improve the interaction with the surrounding matrix but is connected to difficult and expensive treatments, which are usually inconvenient for industrial applications. Here, a simple and eco-friendly method is presented for the oxidation of CNT, where hydrogen peroxide (H_2_O_2_) is the only chemical needed and no toxic emissions are released. Also, the extensive step of the incorporation of CNT to an epoxy matrix is simplified to an ultrasonic dispersion in the liquid hardener component. The effectiveness is proven by mechanical tests of produced CNT/CFRP and compared to a conventional processing route. The combination of those simple and cost efficient strategies can be utilized to produce multiscale composites with improved mechanical performance in an ecological and economical way.

## 1. Introduction

The use of carbon nanotubes (CNT) as a 1-dimensional filler material in modern composite materials opens up unprecedented opportunities in terms of their mechanical, optical, thermal, and electrical properties. Especially in lightweight construction of mechanically highly stressed structural elements in space and aviation, CNT have been studied intensively as unique reinforcement phase. Due to their structural design, CNT are known as the strongest material with a Young’s modulus of 1 TPa [1] and tensile strength of more than 60 GPa [2]. However, the usability of these mechanical properties in composites strongly depends on the transferability of forces between matrix and CNT at the interface and thus is limited by the connection to the matrix. The chemical surface modification of CNT (functionalization) has emerged as an excellent way to establish this connection [3,4,5]. However, current procedures for surface functionalization of CNT are rather expensive and time consuming and therefore hardly commercially lucrative for industrial use.

In research, many different methods have been studied in order to functionalize single-walled (SWCNT) or multi-walled (MWCNT) carbon nanotubes [6,7,8]. In general, these approaches can be divided into two large categories: Firstly, covalent modifications, providing a strong connection to the graphitic surface of the CNT by a chemical (covalent) binding and secondly, non-covalent modifications, exhibiting weaker interaction to the CNT, mainly due to van-der-Waals forces and π-π interactions. Non-covalent functionalization processes are primarily in use when it comes to electrical, thermal, or catalytic applications of CNT. Another non-covalent method involves helical wrapping of the CNT with a polymer [9]. Applications in mechanically stressed structural parts usually benefit from the covalent modification approach. A proper load transfer between matrix and CNT during mechanical stresses of the nanocomposite can be ensured by a chemical linkage due to functionalization, resulting in covalent binding of side-groups from the CNT to the matrix [3]. Hence, functional groups have to be introduced, providing sufficient reactivity to interact with the surrounding matrix.

### 1.1. Oxidation of CNT

Oxidative treatments of CNT usually generate hydroxyl (-OH), carbonyl (-C=O), or carboxyl groups (-COOH) on the surface, which can further react in order to introduce amine functionalities (-NH_2_) for example. Generally, many approaches and syntheses known in chemistry may be used, depending on desired properties of the CNT.

A widely used method is a wet-chemical reaction with oxidizing agents. HNO_3_ [10] or mixes of HNO_3_/H_2_SO_4_ [11] have been studied to generate a reactive surface of the CNT. From graphite chemistry, other chemicals are known for the oxidation of graphitic structures as mixtures with KMnO_4_ [12,13] or KClO_3_ [14,15]. Acidic and basic piranha solutions of sulfuric acid or ammonium hydroxide with hydrogen peroxide (H_2_SO_4_/H_2_O_2_ and NH_4_OH/H_2_O_2_) revealed less oxidation potential for MWCNT compared to HNO_3_ [16]. It was shown that H_2_O_2_ can also be used to oxidize MWCNT by continuous addition of fresh H_2_O_2_ solution over several days [17]. Furthermore, H_2_O_2_ is commonly used for the production of activated carbon (AC) in catalysis [18]. Although, there are certain similarities to carbon black, the procedures cannot be transferred directly to CNT, since they are optimized to achieve controlled microporosity [19] and preservation of mechanical properties is not intended.

One disadvantage of wet-chemical oxidation methods is the related introduction of contaminants, which must be removed afterwards by time intensive washing and filtration steps. However, on the positive side, impurities as amorphous carbon or metal catalyst particles, which occur as byproducts in the synthesis of CNT, are also removed. Therefore, an additional cleaning effect can be achieved due to this treatment. One drawback of covalent functionalization in general is the generation of defects in the graphitic structure of the CNT that means a reduction in its physical properties, such as bulk-, shear-, and Young’s modulus, tensile strength, or electrical and thermal conductivity [20,21,22,23].

As an alternative to wet-chemical processes, CNT can be oxidized via gas phase reactions. Therefore, oxygen has to be transformed into a sufficiently reactive state considering several options. For example, a stream of air or pure oxygen at higher temperatures (>700 °C) may be used for this purpose. However, the yield of CNT is limited due to the formation of CO_2_ and CO [24,25]. Initial studies have been carried out to achieve purification of CNT.

A successful approach of gas phase oxidation of CNT at room temperature is to use ozone (O_3_) as reactive species [26]. Further developments of this method apply a combination of ozone and water vapor (O_3_/H_2_O) [27], indicating that the formation of radical species increases the effectiveness of the reaction by the presence of water vapor. Further, it is possible to combine UV radiation and ozone (UV/O_3_) to generate reactive groups on the surface of a CNT [28]. The absorption of short-wave UV light leads to dissociation of oxygen molecules and thus increases reactivity significantly.

Another approach is to utilize the plasma state in which gas is in an ionized state. First attempts to oxidize MWCNT with an oxygen-plasma were performed to examine changes of the electronic density of states (DOS) of CNT by different oxidation processes [29].

Ozone oxidation reactions of CNT can be performed under atmospheric pressure and room temperature in contrast to plasma processes, which commonly need vacuum conditions and high temperatures. Generally, gas phase oxidation reactions do not require any kind of washing, filtration, or separation steps at all. However, drawbacks of those methods are the poor scalability and low yields, leading to higher product prices.

In summary, the oxidation methods mentioned include several challenges and disadvantages. Low yields, expensive equipment, extensive reactions with high amounts of chemicals, or further steps of purification represent just some of them.

### 1.2. CNT-Reinforced Composites

Nanocomposites of epoxy resin and different amine-functionalized CNT have been investigated as reinforced matrix material [30] for the production of fiber reinforced polymers that are based on carbon fibers [31,32] and glass fibers [33]. Primary amino groups, attached to the CNT, may react with epoxy groups of the resin similar to the curing reaction with amine hardeners [3,34]. However, it was also shown that for polymer nanocomposites, containing CNT concentrations below 1 wt%, the enhancement of tensile strength and Young’s modulus is very similar for oxidized and amine-functionalized CNT [35]. Improvements of different properties of CNT-reinforced composites are still challenging and sometimes limited in comparison to theoretical predictions [36]. Good dispersion of CNT plays a key role, as well as proper functionalization, generating a minimum of structural defects [37].

The ease of dispersion of CNT in various matrices can also be greatly affected by functionalization, thus potentially yielding a combined advantage or disadvantage for composite production. This is particular important for epoxy matrices, where CNT dispersion is typically a great challenge and is often done on a calender, commonly known as three-roll mill (TRM), in a time consuming process, involving several repeat steps. A facile, inexpensive CNT functionalization route combined with an easy to use dispersion approach would therefore greatly impact the possibilities of industrial use.

In the present study, an oxidation treatment of MWCNT is presented that is based only on H_2_O_2_ used in a facile, environmentally benign and inexpensive process. XPS measurements show the effectiveness of different oxidation conditions in comparison to a conventional approach using HNO_3_. Benefits of the oxidation with H_2_O_2_ are subsequently demonstrated by including them in CFRP. In order to avoid time consuming dispersion of CNT in resin on a TRM, an alternative approach of dispersing oxidized CNT in the liquid hardener component by sonication was tested. We show that our simple functionalization and dispersion method yields superior mechanical properties of the composite when adding H_2_O_2_ oxidized MWCNT to the hardener of epoxy matrix as compared to adding untreated MWCNT to the resin by calendering. Our alternative route provides a novel method for effective, eco-friendly oxidation and less elaborative processing of MWCNT for composite applications, besides already existing and more conventional approaches.

## 2. Materials and Methods

### 2.1. Oxidation of CNT

**Figure 1:**
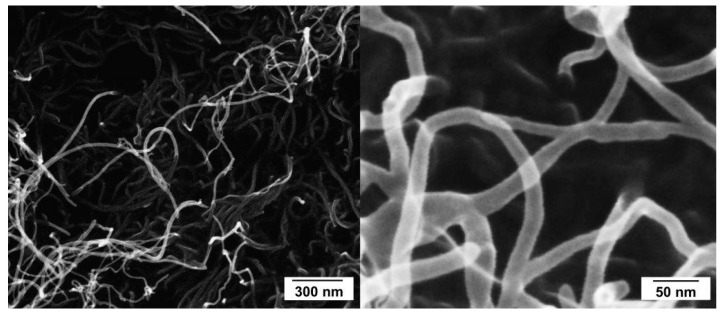
SEM images of used multi-walled carbon nanotubes (MWCNT).

An industrial grade of CVD grown MWCNT obtained from Nanocyl (NC7000, Nanocyl SA., Sambreville, Belgium) was used for the oxidation experiments. According to the datasheet of the supplier, they are characterized by an average diameter of 9.5 nm and 1.5 µm length with a purity of 90% carbon from thermogravimetric analysis [38] and 98.5 at% C analyzed by XPS measurement in a characterization study [39], respectively. SEM images in Figure 1 give a general idea of the dimensions of the used MWCNT.

**Figure 2:**
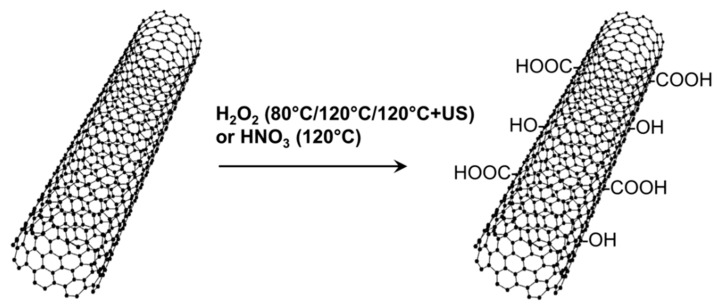
Different oxidation treatments for MWCNT based on H_2_O_2_ or HNO_3_.

Two different oxidation treatments for MWCNT, one based on H_2_O_2_ and the other based on HNO_3_, were investigated for comparison purposes (Figure 2).

The MWCNT were treated in a stabilized 30 wt% aqueous H_2_O_2_ solution (obtained from Carl Roth GmbH + Co. KG, Karlsruhe, Germany) at 80 °C (“CNT-H_2_O_2_, 80 °C”) and 120 °C (“CNT-H_2_O_2_, 120 °C”), respectively. For 200 mg MWCNT, 170 mL H_2_O_2_ solution was used in order to provide an excess of oxidant. The oxidation treatments were carried out for 4 h in a 400 mL round bottom flask under reflux cooling. For a separated amount of MWCNT that were oxidized at 120 °C for 4 h, an ultrasonic treatment in 30 wt% H_2_O_2_ solution was applied for additional 4 h (“CNT-H_2_O_2_, 120 °C + US”). For this purpose, an Elmasonic S10 device (Elma Schmidbauer GmbH, Singen, Germany) with a frequency of 37 kHz was used. For comparison with a conventional method, 150 mg MWCNT were oxidized in 300 mL of a 68 wt% HNO_3_ solution (obtained from Carl Roth GmbH + Co. KG, Karlsruhe, Germany) for 3 h at 120 °C (“CNT-HNO_3_, 120 °C”) in the same equipment.

After the oxidation step, the MWCNT suspension was filtered with a Teflon filter (Zitex G110, retention range: 1–3 µm, thickness: 200–300 µm, diameter: 90 mm, obtained from Carl Roth GmbH + Co. KG, Karlsruhe, Germany) and washed with distilled water. The product was then dried at 70 °C and 80 mbar in a vacuum oven for 24 h.

### 2.2. X-ray Photoelectron Spectroscopy (XPS)

XPS signals were recorded using a Thermo Scientific K-Alpha XPS system (Thermo Fisher Scientific Inc., East Grinstead, UK) equipped with a microfocused, monochromatic Al Kα X-ray source (1486.68 eV). An X-ray beam of 400 µm size was used at 6 mA × 12 kV. The spectra were acquired in the constant analyzer energy mode with pass energy of 200 eV for the survey. Narrow regions were collected with pass energy of 50 eV. Charge compensation was achieved with the system flood gun that provides low energy electrons (~0 eV) and low energy argon ions (20 eV) from a single source. The Thermo Scientific *Avantage* software, version 5.981 (Thermo Fisher Scientific Inc., East Grinstead, UK), was used for digital acquisition and data processing. Spectral calibration was achieved by using the automated calibration routine and the internal Au, Ag and Cu standards supplied with the K-Alpha system. The surface compositions (in at%) were determined by considering the integrated peak areas of the detected atoms and the respective sensitivity factors. The fractional concentration of a particular element A was computed using:

(1) $$\%\;A=\frac{I_{A}/s_{A}}{\sum \left(I_{n}/s_{n}\right)}\times 100\%$$

where $I_{n}$ and $s_{n}$ are the integrated peak areas and the Scofield sensitivity factors corrected for the analyzer transmission, respectively. For the Al Kα source, we used Scofield factors according to Table 1.

The fitting algorithm of Powell was used to fit the XPS peaks. The line shapes were product of Lorentz/Gauss mix and was fixed at L/G = 90%. This value was taken from the fit of C1s peak of pure MWCNT, where we leave to vary also the parameters of shape asymmetry by Powel algorithm. These asymmetry parameters (tail mix, tail height, tail exponent) were then used for sp^2^ signal fitting.

For the measurement, samples were poured on double-sided Cu tape and placed on Al foil. The surface was smoothened by pressing with cleaned Al foil in order to get the surface as smooth as possible without any additional surface contamination. There was no additional cleaning of the sample surface applied (no etching) to ensure the sample was not altered.

### 2.3. Raman Spectroscopy

Raman spectra were measured with a Horiba Jobin Yvon LabRAM HR800 Raman spectrometer (Horiba Europe GmbH, Dresden, Germany) under ambient conditions using a laser with a wavelength of 632.8 nm at 0.75 mW power and a spectral resolution of ~2 cm^−1^. A 50× objective lens of an Olympus optical microscope (Olympus Austria GmbH, Vienna, Austria) was used to focus the beam and collect the signal. For each Raman spectrum, 10 measurements were collected for 30 s from 1000–3000 cm^−1^. Experimental data was smoothed using a cubic spline function.

### 2.4. Dispersion

The used epoxy resin was based on BADGE (Biresin CR170, component A, Sika GmbH, Stuttgart, Germany) with an amine hardener (Biresin CH170-3, component B, Sika GmbH, Stuttgart, Germany). The mass ratio for a mixture of both components is 100:16 (A:B). The MWCNT were dispersed in two different ways, described in the sections below.

#### 2.4.1. Three-Roll Mill

The dispersion of neat MWCNT in epoxy resin was done on a three-roll mill (TRM, Exakt 80E, Exakt Advanced Technologies GmbH, Norderstedt, Germany). Pre-dispersed material of 0.7 wt% CNT in epoxy resin (component A) from a mechanical stirrer was processed within four steps starting from the biggest gap size of 120 µm. In each step, the gap size of both gaps was reduced in order to increase the dispersion quality. Finally, the minimum gap size was applied by setting a line pressure of 10 N/mm between two adjacent rolls.

#### 2.4.2. Ultrasonic

The required amount of filler material was dispersed in the liquid hardener (component B) in order to achieve a concentration of 0.7 wt% oxidized MWCNT (“CNT-H_2_O_2_, 120 °C”), based on the necessary amount of resin, according to the mixing ratio. Hardener and fillers were sonicated in a 400 mL beaker for 30 min.

### 2.5. Preparation of CNT-Reinforced Composites

In order to assess the effectiveness of our H_2_O_2_ treatment, we prepared composites with neat MWCNT and H_2_O_2_-oxidized MWCNT. The HNO_3_-oxidized MWCNT were not used because of the higher defect density and this method has already been investigated extensively in other studies [40,41,42,43].

Resin and hardener were mixed under vacuum conditions of around 100 mbar in a closed glass vessel to avoid the introduction of air bubbles. An electrically driven stirrer with a Teflon blade was used at a rotation speed of 400 rpm for 5 min. For the production of composites, neat MWCNT and only one type of oxidized MWCNT (“CNT-H_2_O_2_, 120 °C”) was used. In case of dispersion on a TRM, the resin contained neat MWCNT (component A + CNT) and the hardener was pure, whereas for ultrasonic dispersion, the hardener contained the mentioned type of oxidized MWCNT (component B + CNT) and the resin was pure.

**Figure 3:**
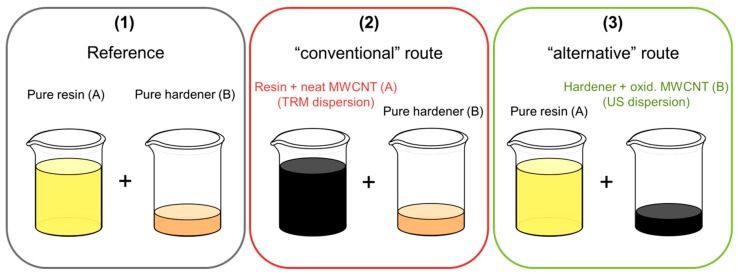
Scheme of different mixes of resin and hardener for the production of carbon fiber reinforced polymer (CFRP).

Carbon fiber reinforced composites (CFRP) were prepared using a plain weave with an areal weight of 245 g/m^²^ and a yarn fineness of 200 tex (3k) warp/weft from high tenacity (HT) carbon fibers (SGL CARBON SE, Wiesbaden, Germany). The CFRP was cured in a heated press at 140 °C for 1 h at 30 bar. Plates with a final thickness of 4 mm were produced using 20 layers of plain weave. Three different mixes for the production of CFRP plates were prepared (Figure 3): a reference that contains no CNT (1), a “conventional” route, using a dispersion of neat MWCNT/epoxy from the TRM (2) and the “alternative” route, using ultrasonic dispersion of oxidized MWCNT in hardener (3).

Testing specimens were cut out of the CFRP plates with a water jet. For the four-point-bending test the geometry of the specimen was 80 × 15 × 4 (mm^³^) according to DIN EN ISO 14125 and the tensile test specimen had a “dog-bone” shape (type 1B) with a total lengths of 170 mm and 20 mm maximal width with a thickness of 4 mm (DIN EN ISO 527-4). The fiber volume content of tested specimens was between 61–63 vol%.

### 2.6. Mechanical Characterization of CNT-Reinforced Composites

The four-point-bending tests were performed on a spindle-driven frame universal testing machine (10 kN, ZwickRoell GmbH & Co. KG, Ulm, Germany). The support span of the fixture had a distance of 66 mm and the loading span distance was 22 mm. Rolls with a diameter of 10 mm were used and the loading speed was set constant to 0.5 mm/min. A digital image correlation (DIC) system was used (Q400, Dantec Dynamics A/S, Skovlunde, Denmark) in order to monitor the deformation of the specimen. For image processing, Istra 4D (V4.4.4.694) was used to determine the flexural modulus.

For tensile tests, the specimens were fixed in hydraulic grips of a Zwick/Roell (100 kN, ZwickRoell GmbH & Co. KG, Ulm, Germany) testing machine. The loading speed was set 2 mm/min and a mechanical extensometer was used for strain recording. Separately, a laser extensometer was used for the evaluation of the Poisson’s ratio. According to the standard, at least five specimens were tested for each series for all tests; except for tensile tests of CFRP that contained pristine MWCNT, only four specimens were tested.

## 3. Results and Discussion

### 3.1. XPS Measurements

**Figure 4:**
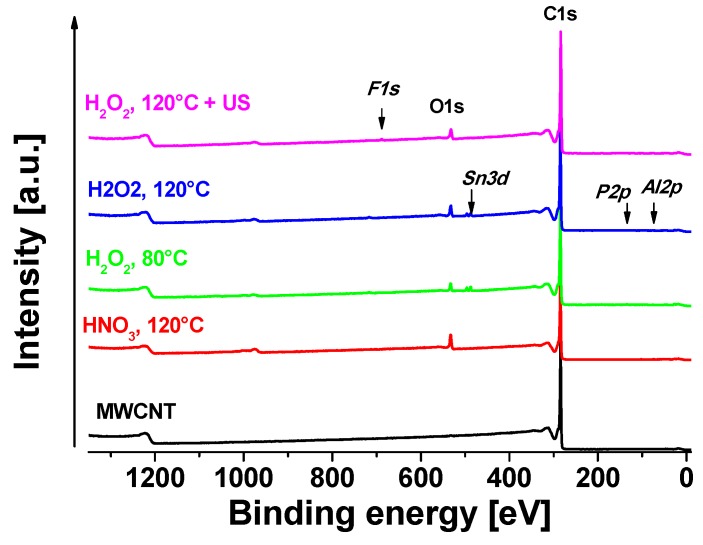
XPS survey of studied samples.

All oxidative treated MWCNT samples were analyzed by XPS in order to compare resulting oxidation degrees. The neat MWCNT were used as reference to oxidized samples. Figure 4 shows the XPS survey of all studied samples. One oxidation strategy was based on H_2_O_2_ at different temperatures (80 °C/120 °C). A separated batch was first oxidized at 120 °C and additionally treated in H_2_O_2_ solution under sonication at room temperature (120 °C + US). In the second approach, HNO_3_ was used as oxidant, which is a well-established method [10,40,44,45].

**Figure 5:**
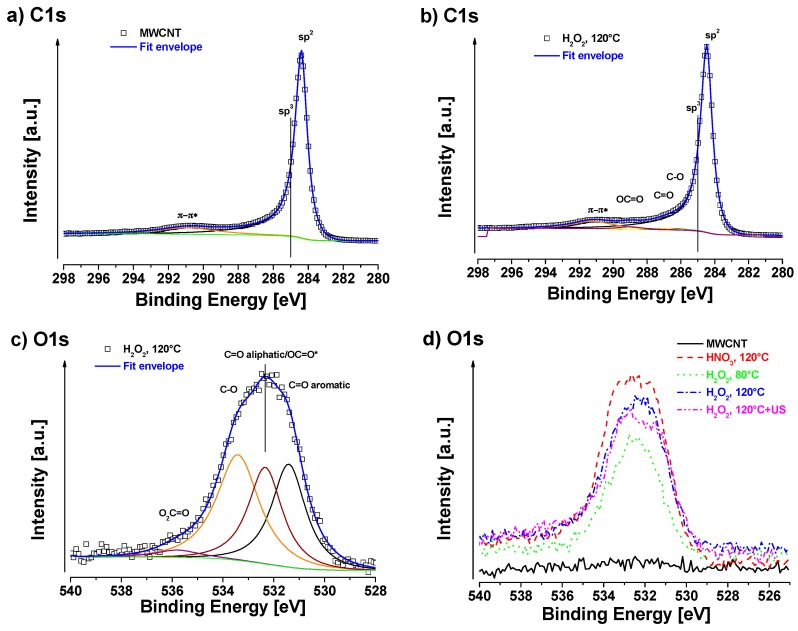
XPS of the (**a**) C1s region of pure MWCNT; (**b**) C1s region of H_2_O_2_, 120 °C; (**c**) O1s region of H_2_O_2_, 120 °C; and (**d**) O1s comparison of prepared samples.

Apparent surface chemical compositions of studied samples are summarized in Table 2. Neat MWCNT only show a negligible amount of oxygen on the surface of about 0.1 at%. C1s signal of neat MWCNT exhibits strong asymmetry because of the sp^2^ peak and the presence of delocalized π-electrons (conduction electrons), available for shake-up like events following core electron photoemission [46]. As was mentioned in the experimental section, we have identified these asymmetry parameters during fitting of neat MWCNT and we used them for fitting of sp^2^ signal for all samples. C1s fit of neat MWCNT sample is shown in Figure 5a and the associated quantification is displayed in Table 2. For oxidized samples, signals of sp^2^ carbon (centered at ~284.4 eV), sp^3^ carbon (~285.0 eV), C–O (~286.4 eV), C=O (~287.1 eV), OC=O (~ 289.2 eV), and π–π* shake-up (~291.2 eV) were detected. The highest oxygen content (~8.5 at%) was detected in the case of MWCNT treated with HNO_3_ at 120 °C. In this case, a certain amount of sp^3^ carbon was also present, indicating some defects in sp^2^. In the case of H_2_O_2_ at 80 °C treatment, the oxygen content was the lowest ca. 3.2 at%. At higher temperature of 120 °C the oxygen content was slightly higher ca. 4.7 at% (sample H_2_O_2_, 120 °C). After ultrasonication of the H_2_O_2_, 120 °C sample, the oxygen slightly decreased to 4.3 at%, which can be consequence of some cleaning of the surface by this procedure, but the decrease is only marginal and practically within the accuracy of XPS (ca. ±10% of particular signal). The chemistry of oxygen species is similar in all cases of oxidized samples, where carbonyls (C=O at ~531.6 eV), carboxyls/esters (OC=O at ~532.5 eV), hydroxyls/ethers (C–O at ~533.5 eV) and some more complicated structures such as O_2_C=O at ~534.8 eV were detected [47]. In the case of the H_2_O_2_-treated samples, there was no sp^3^ carbon fitted, indicating very low amount of defects in sp^2^ structure.

In some samples, a small amount of certain impurities were detected (SnO_2_:Sn3d at ~487.8 eV, phosphates:P2p at ~ 134.5 eV, Al_2_O_3_:Al2p at ~ 75.3 eV, Teflon:F1s at ~ 689.3 eV) coming probably from the processing of the samples. The Al_2_O_3_ could be residues of catalyst from the CVD process and Teflon was used as filter material. Other impurities may come from the H_2_O_2_ solution or water from the washing step but cannot be assigned clearly.

Since there was no beneficial effect from the ultrasonic treatment, the oxidized MWCNT treated at 120 °C without additional ultrasonic were used for further processing in composites. Detailed C1s and O1s fits are presented in Figure 5b,c. Oxidation of the MWCNT was achieved with almost 5 at%, which was desired in order to prevent damage of the structure and retain most of the mechanical properties. The amount of defects that corresponds to sp^3^ carbon is even negliglible in this case. It is well known that the defect density of CNT has a direct influence on their mechanical properties. It is therefore important to keep the balance between functionality from reactive side groups and mechanical properties, given by the intact structure. The critical concentration of carboxylation, which was found to be in the range of 5–6% for a SWCNT, should not be exceeded [20].

A comparison of O1s scans is shown in Figure 5d. A higher degree of oxidation was observed at higher temperatures for H_2_O_2_. However, the oxidation effect of HNO_3_ was more pronounced than for H_2_O_2_ at the same tempertature (120 °C). The oxidation potential of CNT in water was found to be ~0.80 V [48] and the standard potentials of used oxidants are 0.96 V and 1.77 V for HNO_3_ and H_2_O_2_, respectively [49]. That means that, thermodynamically, the oxidation reaction of CNT should be possible with both oxidants, according to the following reactions:

4 CNT (reduced) + O_2_ + 4 H^+^ → 4 CNT^+^ (oxidized) + 2 H_2_O; 0.80 V (2)

H_2_O_2_ + 2 H^+^ + 2e^−^ → 2 H_2_O; 1.77 V (3)

HNO_3_ + 3 H^+^ + 3e^−^ → NO + 2 H_2_O; 0.96 V (4)

According to the Nernst equation, the potential also depends on temperature and the ratio of concentrations of oxidized to reduced species, which means an increased oxidation potential at higher temperature and oxidant excess.

For H_2_O_2_, there is also an autocatalytic decomposition reaction involved that is accelerated at increased temperature.

2 H_2_O_2_ → 2 H_2_O + O_2_ (5)

At higher temperatures around 110–120 °C, H_2_O_2_ becomes unstable and decomposes spontaneously to hydroxyl radicals and perhydroxy radicals [50].

H_2_O_2_ → 2 HO^•^ (6)

H_2_O_2_ → HOO^•^ + H^•^ (7)

These radicals are considered to react with the surface of CNT in aqueous solution (radical scavenging), leading to their oxidation [51,52].

2 CNT + 2 HO^•^ → 2 CNT^+^ + O_2_ + H_2_ (8)

The type of appearing functional groups on the surface of the CNT by a H_2_O_2_ oxidation depends on the detailed conditions of the treatment. For example, only hydroxyl functionalization was detected by FTIR measurements after autoclaving and sonication of CNT in 30% H_2_O_2_ solution [53]. Another study reports about highly oxidized MWCNT by 30% H_2_O_2_ over several days at 65 °C by adding fresh H_2_O_2_ in certain intervals that led to the presence of hydroxyl, carbonyl, and carboxyl functionalization in XPS analysis [17]. A quite long reaction time was necessary because of the low temperature that was applied. In contrast, we used elevated temperature in order to minimize oxidation time and effort of the functionalization treatment, by generating higher amounts of radicals. Further, the reaction conditions were optimized for a final oxygen content of around 5 at% to avoid excessive structure damage on the one hand and provide functioinal goups that reduce agglomeration and interact with the matix on the other hand.

### 3.2. Raman Spectroscopy

**Figure 6:**
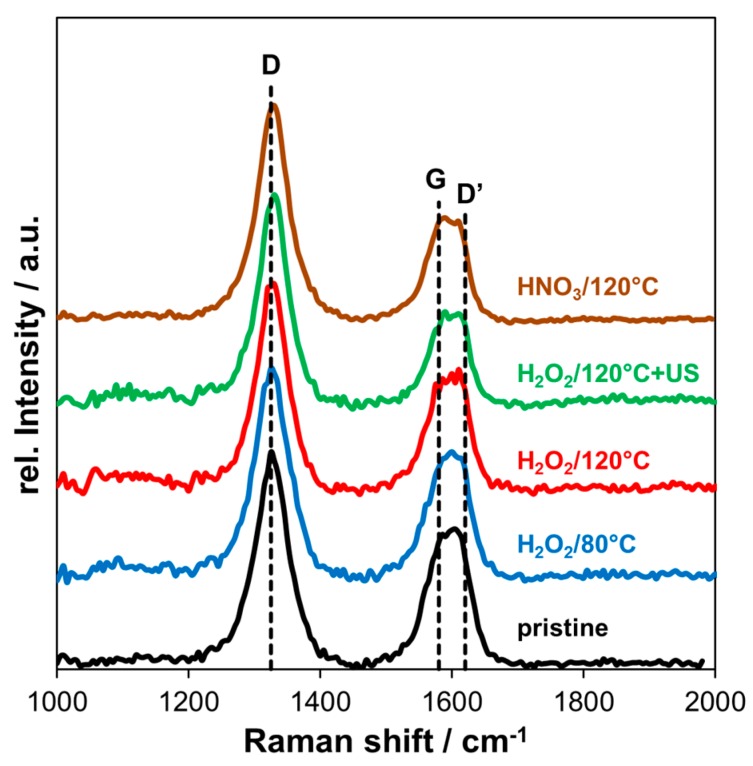
Raman spectra of pristine and oxidized MWCNT using different oxidative treatments.

In Figure 6, the Raman spectra of pristine and oxidized MWCNT are shown. The D band (disorder) is located at ~1325 cm^−1^ and the G band (graphitic) at ~1580 cm^−1^. Further, a D’ band is present at higher frequency next to the G band at ~1620 cm^−1^.

Covalent functionalization of graphitic structure corresponds to an increase in defect density due to the generation of sp^3^ carbon. Hence, the ratio between intensity of the D band and the G band (*I_D_/I_G_*) was used to indicate structural damage by the oxidation. For pristine MWCNT, the ratio *I_D_/I_G_* was 1.61, increasing to 1.72 and 1.81 for H_2_O_2_ oxidation at 80 °C and 120 °C, respectively. After applying additional ultrasonic treatment in H_2_O_2_ after oxidation the ratio increased to 2.21 and the oxidation with HNO_3_ at 120 °C led to an *I_D_/I_G_* ratio of 2.08.

Comparing the *I_D_/I_G_* ratios shows that oxidation with H_2_O_2_ at both temperatures only slightly increased the amount of defects/disorder compared to pristine MWCNT. In contrast, ultrasonic treatment and HNO_3_ oxidation damaged the graphitic structure significantly. In XPS measurements, additional sp^3^ carbon was detected after HNO_3_ oxidation and also the amount of oxygen was higher (8.5 at%), indicating that structural damage was mainly caused by covalent attachment of functional groups. For additional ultrasonication after H_2_O_2_ oxidation of MWCNT, the oxygen content did not further increase. However, the amount of defects is clearly more pronounced in Raman measurements. This could be explained by the shortening effect of MWCNT due to ultrasonic treatment, which generates open edge sites. Amorphous carbon from the inside of the tube could be released in this way or directly generated on the surface of the outer wall, which was reported in the literature [54]. Especially, this is relevant for relatively long sonication time of 1 h in H_2_O_2_ solution, in our case.

In general, the D band in the Raman spectra of sp^2^ carbon is attributed to disordered carbon, namely hetero-atoms, vacancies, grain boundaries, amorphous carbon, or any other finite size structures which lower the crystalline symmetry [55]. In fact, for MWCNT, an exact correlation between individual contributions of specific defects and the corresponding Raman signal has not been established yet [56].

### 3.3. Dispersion of MWCNT

**Figure 7:**
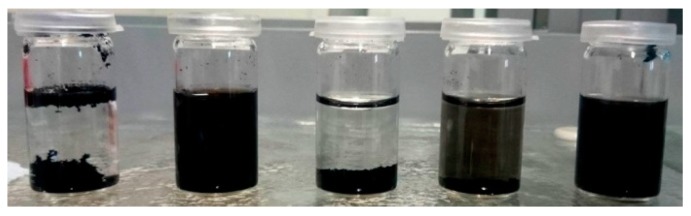
Dispersion of different CNT in water (from left to right): neat MWCNT, oxidized MWCNT in HNO_3_ (120 °C), H_2_O_2_ (80 °C), H_2_O_2_ (120 °C), and H_2_O_2_ (120 °C + US treatment), respectively.

As a first evaluation of the successful oxidation of CNT, their dispersion behavior in distilled water can be investigated. A higher degree of oxidation will lead to improved dispersion due to repulsive forces by dissociated groups. In Figure 7, dispersions of MWCNT, neat and after different oxidative treatments, in water are shown after 30 min of sonication and 1 h sedimentation. It can be observed that after constant sedimentation time the oxidation with HNO_3_ and H_2_O_2_ at 120 °C led to improved dispersion behavior, whereas neat MWCNT separated on bottom and top. Oxidation with H_2_O_2_ at 80 °C only slightly increased the dispersibility of treated MWCNT in water. These results also indicate that dispersion in the polar hardener component is possible for oxidized CNT but not for neat CNT, which naturally exhibit an entire hydrophobic surface.

**Figure 8:**
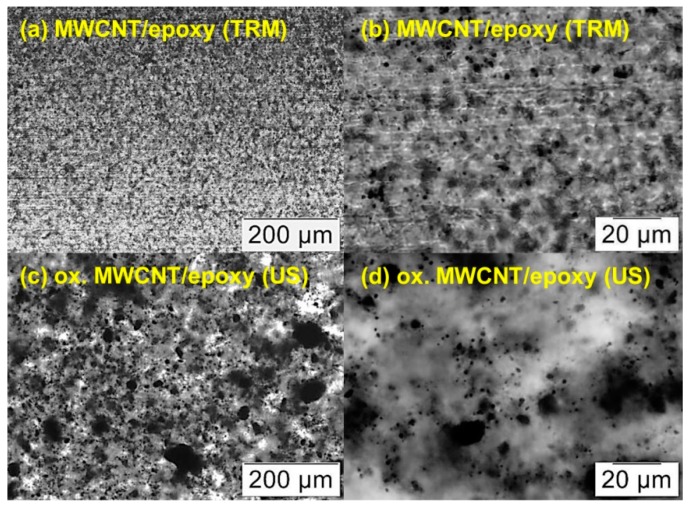
Light microscope images of section cuts of cured CNT/epoxy samples containing 0.7 wt% CNT related to the resin: (**a**,**b**) show neat MWCNT, dispersed in the resin with a three-roll mill (TRM) and mixed with the hardener by a mechanical stirrer. (**c**,**d**) show oxidized MWCNT, which were dispersed with ultrasonic (US) in the hardener first and mixed with the epoxy resin using a mechanical stirrer.

The dispersion of cured CNT/epoxy nanocomposites was compared by investigation of light microscope images of section cuts (Figure 8). The “conventional” route involved dispersing neat MWCNT by a calender (three-roll mill, TRM), while our more straightforward “alternative” route was based on ultrasonic mixing of H_2_O_2_ oxidized MWCNT into the hardener (no TRM step required) In general, the calender dispersion technique led to more homogenous distribution and smaller agglomerates of MWCNT with a maximum of approximately 5–10 µm in diameter (Figure 8a,b). In this case, the neat MWCNT were directly dispersed in epoxy resin (component A) which makes up the main part of the mixture. Dispersing oxidized MWCNT in the amine hardener (component B) with ultrasonic shows a larger variety of agglomerate size from several microns up to around 100 µm (Figure 8c). However, most of the large agglomerates are composed of small ones which are located close to each other (Figure 8d).

To ensure the successful reinforcement of the epoxy matrix by oxidized MWCNT, tensile tests were performed. The results showed an enhancement of Young’s modulus from 2750 MPa to 3700 MPa and increase of tensile strength from 41 MPa to 48 MPa for neat epoxy and MWCNT/epoxy nanocomposites, respectively. It should be mentioned that some properties of the matrix are changing due to the addition of MWCNT, which were not investigated in this study. The electronical and thermal conductivities usually increase [57], while the glass transition temperature (T_g_) drops compared to unfilled epoxy resin [58].

### 3.4. Mechanical Testing of CNT-Reinforced CFRP

In four-point-bending tests of CFRP, the addition of both types, neat MWCNT and oxidized MWCNT, to the epoxy matrix led to improvements of flexural modulus and strength compared to the reference that was produced without CNT (Table 3). Neat MWCNT that were dispersed in the resin with a TRM increased the modulus and strength of CFRP by +10% and +12%, respectively. Significantly higher results were achieved by the dispersion of oxidized MWCNT in the hardener component, increasing the modulus of tested CFRP specimens by +22% and by +56% in flexural strength. This is particularly remarkable, since our oxidized MWCNT were—due to the simpler dispersion approach—less perfectly dispersed. Nevertheless, this fact was obviously outweighed by the better interaction of matrix and filler, which led to higher mechanical performance.

**Figure 9:**
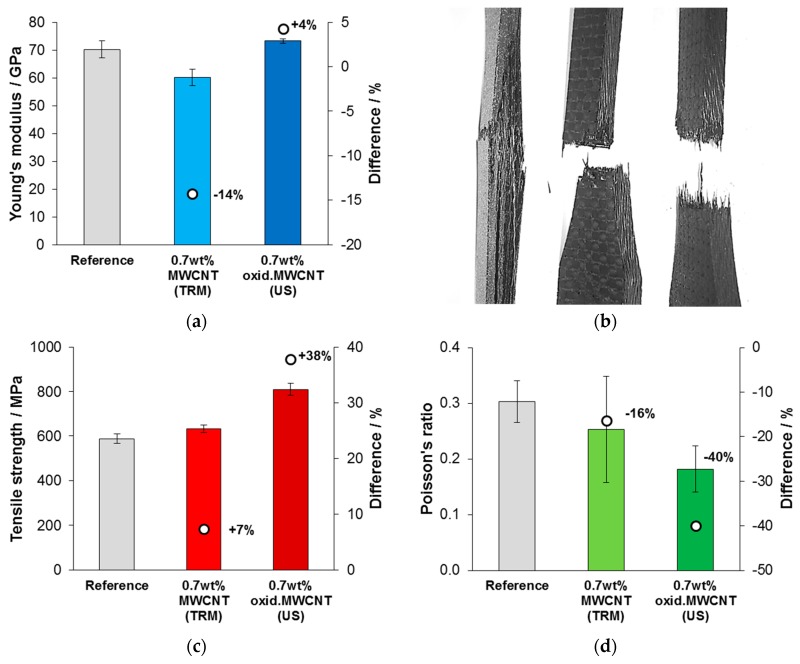
Results of tensile tests of CFRP: (**a**) Young’s modulus, (**b**) images of tested CFRP (reference, filled with MWCNT, filled with oxidized MWCNT) with their typical fracture behavior, (**c**) tensile strength and (**d**) Poisson’s ratio.

The results of tensile tests are presented in Figure 9. A reduction in the Young’s modulus for CFRP that were modified with neat MWCNT was found, whereas the modulus did not change for oxidized MWCNT modification within a statistical significance, compared to the reference (Figure 9a). Obviously, a different failure mechanism of the tensile specimens occurred when MWCNT were added, as it is shown in Figure 9b. Strong delamination was observed for specimens with unmodified matrix. In contrast, tensile specimens that were modified with neat or oxidized MWCNT stayed compact after failure. Figure 9c shows that the tensile strength was slightly improved by adding neat MWCNT (+7%) but much more effectively by oxidized MWCNT (+38%). Mean values for the Poisson’s ratio, measured in thickness dimension of the specimen (4 mm), were different for each series of tested CFRP specimens (Figure 9d). The reference showed the highest Poisson’s ratio and a reduction was observed by adding neat MWCNT (−16%) and oxidized MWCNT (−40%).

Less transversal contraction of the composite under tensile load—which is expressed by the Poisson’s ratio—could be the reason for different delamination behavior, since the CNT stiffen the matrix. Due to a reduced delamination of the specimen, increased values for strength under tensile and bending load could be derived.

**Figure 10:**
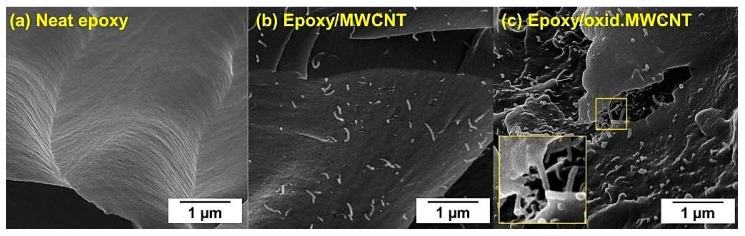
SEM images of fracture surface of (**a**) neat epoxy and epoxy/CNT nanocomposites containing (**b**) pristine MWCNT and (**c**) oxidized MWCNT.

The results also underline the importance of surface functionalization of CNT for efficient improvement of mechanical properties of nanomodified composites. In case of ultrasonic dispersion of oxidized MWCNT in the hardener, covalent bonding between amine groups (-NH_2_) of the hardener and carboxylic groups (-COOH) can be assumed (amidation reaction) during the curing reaction. A connection of the second amine group of the bifunctional hardener molecule to the epoxy resin could establish a direct linkage, creating a network with much more efficient stress transfer from the cured composite to the CNT under mechanical load. Improved stress distribution is expected to be the underlying mechanism for the enhanced mechanical performance of the composites. Figure 10 shows SEM images of neat epoxy and MWCNT/epoxy nanocomposites after tensile failure. The fracture surface of unfilled resin (Figure 10a) is very smooth, whereas the addition of CNT increased the roughness and thereby the specific fracture energy. Individual MWCNT can be identified in Figure 10b,c. Several reinforcement mechanisms have been already reported extensively in the literature, including pull-out, fracture, and crack bridging of CNT [59,60]. Due to enhanced chemical affinity of oxidized MWCNT to the epoxy matrix, a stronger interfacial bonding was achieved, resulting in less CNT pull-out and more efficient crack bridging, which was observed in Figure 10c.

Compared to other studies (see Table 4) we demonstrate a very simple approach for the functionalization and dispersion of MWCNT for the mechanical improvement of CNT-modified carbon fiber/epoxy composites. Besides the eco-friendly aspect of the oxidation treatment, our processing method has proven to be productive and effective. Except for the Young’s modulus, which is dominated by the carbon fibers, tensile and flexural strength as well as flexural modulus were increased significantly. Especially, the flexural strength shows the highest increase in this comparison table, which may be attributed to the very effective improvement of the matrix modulus that stabilized the carbon fibers under bending load and thus reduced the fiber buckling.

Taking into account twice the standard deviation as the lower limit when comparing our alternative route with the reference, the improvement of tested mechanical properties is still of statistical significance, e.g., +9% flexural modulus, +34% flexural strength, and +20% tensile strength, respectively.

## 4. Conclusions

In this study, it was shown that MWCNT can be oxidized efficiently by hydrogen peroxide (H_2_O_2_) at elevated temperature in order to obtain functionalized CNT for composite materials. A functionalization degree of around 5 at% oxygen was found in XPS measurements, without creating substantial amounts of defects, in contrast to oxidation with HNO_3_. Mainly hydroxyl (-OH), carbonyl (-C=O), and carboxyl (-COOH) groups were present after the oxidation treatments. Therefore, the oxidation of CNT with H_2_O_2_ can be considered as a “green” and effective process. An alternative dispersion route of oxidized MWCNT in amine hardener via sonication was presented, revealing a sufficient dispersion quality in cured epoxy resin. However, dispersing untreated MWCNT in the resin on a three-roll mill (TRM) led to a more homogenous distribution. Both dispersion techniques were used for the production of CNT-modified CFRP and their mechanical properties were tested in four-point-bending and tensile tests. Substantial improvements in mechanical performance were obtained by adding oxidized MWCNT using US dispersion in our “alternative” approach and only minor increases by the “conventional” route involving neat MWCNT dispersed on the TRM.

In general, our approach of eco-friendly oxidation of CNT and simple ultrasonic dispersion in hardener could be applied to various CNT/polymer nanocomposites consisting of two-component matrix systems and not only for carbon fiber reinforced composites.

## Figures and Tables

**Table 1:** Scofield factors used for XPS analysis.

C1s	O1s	F1s	Al2p	Sn3d	P2p
1.000	2.881	4.118	0.560	37.257	1.353

**Table 2:** Apparent surface chemical composition of prepared MWCNT samples as determined by XPS measurements.

Sample	Surface Chemical Composition [at%] (Peak Positions in eV)					
	C1s	O1s	Sn3d	P2p	Al2p	F1s
	sp^2^/sp^3^/C-O/C=O/OC=O/π-π*	C=O_ar_/OC=O^x^/C-O/O_2_C=O	SnO_2_	PO_4_	Al_2_O_3_	C-F
	(248.4/285/286.4/287.1/289.2/291.2)	(531.6/532.5/533.5/534.8)	(487.8)	(134.5)	(75.3)	(689.3)
MWCNT	99.9	0.1	—	—	—	—
	100/0/0/0/0/0	—				
HNO_3_, 120 °C	91.5	8.5	—	—	—	—
	82.1/3.7/2.0/0.7/3.6/7.8	33.5/25.3/38.1/3.1				
H_2_O_2_, 80 °C	96.4	3.2	0.2	0.2	—	—
	87.9/0/0.7/0.9/1.0/9.5	29.7/36.0/31.6/2.7				
H_2_O_2_, 120 °C	95.0	4.7	0.1	0.1	0.2	—
	86.9/0/1.0/0.9/1.7/9.6	30.2/28.3/38.8/2.7				
H_2_O_2_, 120 °C + US	95.3	4.3	—	—	0.2	0.3
	87.9/0/1.5/0.2/1.6/8.9	29.4/17.3/46.9/6.4				

**Table 3:** Results from four-point-bending tests of CFRP specimens with different fillers and dispersion techniques. The numbers are mean values and the standard deviation is given in brackets. Improvements are related to reference specimens that do not contain any filler.

Filler Type	Filler Content	Dispersion	Flexural Modulus [GPa]	Change [%]	Flexural Strength [MPa]	Change [%]
Reference	-	-	40.2 (±1.8)	-	520 (±20)	-
MWCNT	0.7 wt%	TRM in resin	44.1 (±2.7)	+10	580 (±45)	+12
oxidized MWCNT	0.7 wt%	US in hardener	49.0 (±0.7)	+22	810 (±30)	+56

**Table 4:** Comparison of different functionalization and dispersion approaches and the resulting improvement of mechanical properties of CNT-modified carbon fiber/epoxy composites. (Young’s modulus, E; tensile strength, σ; flexural modulus, E_f_ and flexural strength, σ_f_; three-roll mill, TRM).

Filler Type	Functionalization	Filler Content	Dispersion	Property	Change	Reference
oxidized MWCNT	H_2_O_2_	0.7 wt%	sonication in hardener	E	+4%	this study
				σ	+38%	
				E_f_	+22%	
				σ_f_	+56%	
oxidized MWCNT	no information	0.3 wt%	sonication and TRM in resin	E_f_	+15%	[61]
				σ_f_	+19%	
oxidized MWCNT	H_2_SO_4_/HNO_3_	1.0 wt%	magnetic stirring in mixture of resin and hardener	E	+16%	[62]
				σ	+7%	
silanized MWCNT	3-aminopropyl-triethoxysilane	1.0 wt%		E	+34%	
				σ	+16%	
MWCNT-NH_2_	no information	0.7 wt%	TRM in resin	E_f_	+13%	[63]
				σ_f_	+18%	
MWCNT-NH_2_	H_2_SO_4_/HNO_3_, SOCl_2_, Diethylamine	1.0 wt%	sonication and ball milling in resin	E	+49%	[31]
				σ	+52%	
				σ_f_	+38%	
MWCNT-NH_2_	oxidation, SOCl_2_, 1,2-ethylene-diamine	0.5 wt%	mechanical stirring and sonication in resin	E_f_	+6%	[32]
				σ_f_	+23%	
MWCNT-NH_2_	oxidation, SOCl_2_, diethyltoluene-diamine	0.5 wt%		E_f_	+18%	
				σ_f_	+37%	

## References

[1] Treacy M.M.J., Ebbesen T.W., Gibson J.M. (1996). Exceptionally high Young’s modulus observed for individual carbon nanotubes. Nature.

[2] Yu M.F., Lourie O., Dyer M.J., Moloni K., Kelly T., Ruoff R. (2000). Strength and Breaking Mechanism of Multiwalled Carbon Nanotubes under Tensile Load. Science.

[3] Wang S., Liang R., Wang B., Zhang C. (2008). Load-transfer in functionalized carbon nanotubes/polymer composites. Chem. Phys. Lett..

[4] Ma P.C., Zheng Q.B., Mäder E., Kim J.K. (2012). Behavior of load transfer in functionalized carbon nanotube/epoxy nanocomposites. Polymer.

[5] Coleman J.N., Khan U., Blau W.J., Gun’ko Y.K. (2006). Small but strong: A review of the mechanical properties of carbon nanotube–polymer composites. Carbon.

[6] Sahoo N.G., Rana S., Cho J.W., Li L., Chan S.H. (2010). Polymer nanocomposites based on functionalized carbon nanotubes. Prog. Polym. Sci..

[7] Karousis N., Tagmatarchis N., Tasis D. (2010). Current Progress on the Chemical Modification of Carbon Nanotubes. Chem. Rev..

[8] Tasis D., Tagmatarchis N., Bianco A., Prato M. (2006). Chemistry of Carbon Nanotubes. Chem. Rev..

[9] Fu H., Xu S., Li Y. (2016). Nanohelices from planar polymer self-assembled in carbon nanotubes. Sci. Rep..

[10] Stobinski L., Lesiak B., Kövér L., Tóth J., Biniak S., Trykowski G., Judek J. (2010). Multiwall carbon nanotubes purification and oxidation by nitric acid studied by the FTIR and electron spectroscopy methods. J. Alloys Compd..

[11] Zhou W., Sasaki S., Kawasaki A. (2014). Effective control of nanodefects in multiwalled carbon nanotubes by acid treatment. Carbon.

[12] Hummers W.S., Offeman R.E. (1958). Preparation of Graphitic Oxide. J. Am. Chem. Soc..

[13] Nanyan Z., Jining X., Vijay K.V. (2002). Functionalization of carbon nanotubes by potassium permanganate assisted with phase transfer catalyst. Smart. Mater. Struct..

[14] Poh H.L., Šaněk F., Ambrosi A., Zhao G., Sofer Z., Pumera M. (2012). Graphenes prepared by Staudenmaier, Hofmann and Hummers methods with consequent thermal exfoliation exhibit very different electrochemical properties. Nanoscale.

[15] Staudenmaier L. (1898). Verfahren zur Darstellung der Graphitsäure. Ber. Dtsch. Chem. Ges..

[16] Datsyuk V., Kalyva M., Papagelis K., Parthenios J., Tasis D., Siokou A., Kallitsis I., Galiotis C. (2008). Chemical oxidation of multiwalled carbon nanotubes. Carbon.

[17] Peng Y., Liu H. (2006). Effects of oxidation by hydrogen peroxide on the structures of multiwalled carbon nanotubes. Ind. Eng. Chem. Res..

[18] Rodríguez-reinoso F. (1998). The role of carbon materials in heterogeneous catalysis. Carbon.

[19] Linares-Solano A., Lozano-Castello D., Lillo-Ródenas M., Cazorla-Amorós D. (2007). Controlling Porosity to Improve Activated Carbon Applications.

[20] Milowska K.Z. (2015). Influence of Carboxylation on Structural and Mechanical Properties of Carbon Nanotubes: Composite Reinforcement and Toxicity Reduction Perspectives. J. Phys. Chem. C.

[21] Salvetat J.P., Bonard J.M., Thomson N.H., Kulik A.J., Forró L., Benoit W., Zuppiroli L. (1999). Mechanical properties of carbon nanotubes. Mater. Sci. Semicond. Process..

[22] Charlier J.C. (2002). Defects in carbon nanotubes. Acc. Chem. Res..

[23] Li W., Feng Y., Peng J., Zhang X. (2012). Effects of Stone-Wales Defects on the Thermal Conductivity of Carbon Nanotubes. J. Heat. Transfer..

[24] Ajayan P.M., Ebbesen T.W., Ichihashi T., Iijima S., Tanigaki K., Hiura H. (1993). Opening carbon nanotubes with oxygen and implications for filling. Nature.

[25] Ebbesen T.W., Ajayan P.M., Hiura H., Tanigaki K. (1994). Purification of nanotubes. Nature.

[26] Mawhinney D.B., Naumenko V., Kuznetsova A., Yates J.T., Liu J., Smalley R.E. (2000). Infrared Spectral Evidence for the Etching of Carbon Nanotubes:  Ozone Oxidation at 298 K. J. Am. Chem. Soc..

[27] Peng K., Liu L.Q., Li H., Meyer H., Zhang Z. (2011). Room temperature functionalization of carbon nanotubes using an ozone/water vapor mixture. Carbon.

[28] Sham M.L., Kim J.K. (2006). Surface functionalities of multi-wall carbon nanotubes after UV/Ozone and TETA treatments. Carbon.

[29] Ago H., Kugler T., Cacialli F., Salaneck W.R., Shaffer M.S.P., Windle A.H., Friend R.H. (1999). Work Functions and Surface Functional Groups of Multiwall Carbon Nanotubes. J. Phys. Chem. B.

[30] Guadagno L., De Vivo B., Di Bartolomeo A., Lamberti P., Sorrentino A., Tucci V., Vertuccio L., Vittoria V. (2011). Effect of functionalization on the thermo-mechanical and electrical behavior of multi-wall carbon nanotube/epoxy composites. Carbon.

[31] Sharma K., Shukla M. (2014). Three-Phase Carbon Fiber Amine Functionalized Carbon Nanotubes Epoxy Composite: Processing, Characterisation, and Multiscale Modeling. J. Nanomater..

[32] Wu J., Guo J., Zhang Q., Gao L., Li H., Deng H., Jiang W., Sui G., Yang X. (2018). Effect of different amino functionalized carbon nanotubes on curing behavior and mechanical properties of carbon fiber/epoxy composites. Polym. Compos..

[33] Garg M., Sharma S., Mehta R. (2015). Pristine and amino functionalized carbon nanotubes reinforced glass fiber epoxy composites. Compos. Part A Appl. Sci. Manuf..

[34] Gojny F.H., Nastalczyk J., Roslaniec Z., Schulte K. (2003). Surface modified multi-walled carbon nanotubes in CNT/epoxy-composites. Chem. Phys. Lett..

[35] Singh B.P., Singh D., Mathur R.B., Dhami T.L. (2008). Influence of Surface Modified MWCNTs on the Mechanical, Electrical and Thermal Properties of Polyimide Nanocomposites. Nanoscale Res. Lett..

[36] Shokrieh M.M., Rafiee R. (2010). A review of the mechanical properties of isolated carbon nanotubes and carbon nanotube composites. Mech. Compos. Mater..

[37] Ma P.C., Siddiqui N.A., Marom G., Kim J.K. (2010). Dispersion and functionalization of carbon nanotubes for polymer-based nanocomposites: A review. Compos. Part A Appl. Sci. Manuf..

[38] Nanocyl SA Technical Data Sheet Nanocyl NC7000. http://www.nanocyl.com/wp-content/uploads/2016/07/DM-TI-02-TDS-NC7000-V08.pdf.

[39] White C.M., Banks R., Hamerton I., Watts J.F. (2016). Characterisation of commercially CVD grown multi-walled carbon nanotubes for paint applications. Prog. Org. Coat..

[40] Rosca I.D., Watari F., Uo M., Akasaka T. (2005). Oxidation of multiwalled carbon nanotubes by nitric acid. Carbon.

[41] Avilés F., Cauich-Rodríguez J.V., Moo-Tah L., May-Pat A., Vargas-Coronado R. (2009). Evaluation of mild acid oxidation treatments for MWCNT functionalization. Carbon.

[42] Phan C.H., Jaafar M., Koh Y.H. (2015). Mild functionalization of carbon nanotubes filled epoxy composites: Effect on electromagnetic interferences shielding effectiveness. J. Appl. Polym. Sci..

[43] Špitalský Z., Krontiras C.A., Georga S.N., Galiotis C. (2009). Effect of oxidation treatment of multiwalled carbon nanotubes on the mechanical and electrical properties of their epoxy composites. Compos. Part A Appl. Sci. Manuf..

[44] Tchoul M.N., Ford W.T., Lolli G., Resasco D.E., Arepalli S. (2007). Effect of Mild Nitric Acid Oxidation on Dispersability, Size, and Structure of Single-Walled Carbon Nanotubes. Chem. Mater..

[45] Andrade N.F., Martinez D.S.T., Paula A.J., Silveira J.V., Alves O.L., Souza Filho A.G. (2013). Temperature effects on the nitric acid oxidation of industrial grade multiwalled carbon nanotubes. J. Nanopart. Res..

[46] Avantage XPS Software Database.

[47] Hantsche H. (1993). High resolution XPS of organic polymers, the scienta ESCA300 database. By G. Beamson and D. Briggs, Wiley, Chichester. Adv. Mater..

[48] Zheng M., Diner B.A. (2004). Solution Redox Chemistry of Carbon Nanotubes. J. Am. Chem. Soc..

[49] Becker H.G.O. (2004). Organikum: Organisch-chemisches Grundpraktikum.

[50] Sundara R. (1998). Hot peroxide bleaching. Can. Chem. News.

[51] Tsuruoka S., Matsumoto H., Castranova V., Porter D.W., Yanagisawa T., Saito N., Kobayashi S., Endo M. (2015). Differentiation of chemical reaction activity of various carbon nanotubes using redox potential: Classification by physical and chemical structures. Carbon.

[52] Tsuruoka S., Matsumoto H., Koyama K., Akiba E., Yanagisawa T., Cassee F.R., Saito N., Usui Y., Kobayashi S., Porter D.W. (2015). Radical scavenging reaction kinetics with multiwalled carbon nanotubes. Carbon.

[53] Weydemeyer E.J., Sawdon A.J., Peng C.A. (2015). Controlled cutting and hydroxyl functionalization of carbon nanotubes through autoclaving and sonication in hydrogen peroxide. Chem. Commun..

[54] Lu K.L., Lago R.M., Chen Y.K., Green M.L.H., Harris P.J.F., Tsang S.C. (1996). Mechanical damage of carbon nanotubes by ultrasound. Carbon.

[55] Brown S.D.M., Jorio A., Dresselhaus M.S., Dresselhaus G. (2001). Observations of the D-band feature in the Raman spectra of carbon nanotubes. Phys. Rev. B.

[56] Lehman J.H., Terrones M., Mansfield E., Hurst K.E., Meunier V. (2011). Evaluating the characteristics of multiwall carbon nanotubes. Carbon.

[57] Gardea F., Lagoudas D.C. (2014). Characterization of electrical and thermal properties of carbon nanotube/epoxy composites. Compos. Part B Eng..

[58] Khare K.S., Khare R. (2013). Effect of Carbon Nanotube Dispersion on Glass Transition in Cross-Linked Epoxy–Carbon Nanotube Nanocomposites: Role of Interfacial Interactions. J. Phys. Chem. B.

[59] Thostenson E.T., Ren Z., Chou T.W. (2001). Advances in the science and technology of carbon nanotubes and their composites: A review. Compos. Sci. Technol..

[60] Spitalsky Z., Tasis D., Papagelis K., Galiotis C. (2010). Carbon nanotube–polymer composites: Chemistry, processing, mechanical and electrical properties. Prog. Polym. Sci..

[61] Islam M.E., Mahdi T.H., Hosur M.V., Jeelani S. (2015). Characterization of Carbon Fiber Reinforced Epoxy Composites Modified with Nanoclay and Carbon Nanotubes. Procedia. Eng..

[62] Lee J.H., Rhee K.Y., Park S.J. (2011). Silane modification of carbon nanotubes and its effects on the material properties of carbon/CNT/epoxy three-phase composites. Compos. Part A Appl. Sci. Manuf..

[63] Singer G., Rennhofer H., Sinn G., Unterlass M.M., Wendrinsky J., Windberger U., Lichtenegger H.C. (2017). Processing of Carbon Nanotubes and Carbon Nanofibers towards High Performance Carbon Fiber Reinforced Polymers. Key Eng. Mater..

